# Practical Notes on the Treatment of Skin Diseases

**Published:** 1886-03

**Authors:** 


PRACTICAL NOTES ON THE TREATMENT OF SKIN DISEASES. Diseases of the Perspiratory and Sebaceous Glands. By George H. Rohé, M. D., Professor of Hygiene and Clinical Dermatology in the College of Physicians and Surgeons. Baltimore, 1885, pp. 59.

This little work, in pamphlet form, is the first of a series which the author intends to publish. The series is to comprise practical discussions of the different diseases of the skin. The number before us contains a concise description of the different affections of the glandular apparatus of the skin and the suggestions as to treatment, though possessing no original features, are precise and worthy of attention.

